# Characteristics of circulating immune cells in HBV-related acute-on-chronic liver failure following artificial liver treatment

**DOI:** 10.1186/s12865-023-00579-8

**Published:** 2023-11-25

**Authors:** Tao Ju, Daixi Jiang, Chengli Zhong, Huafen Zhang, Yandi Huang, Chunxia Zhu, Shigui Yang, Dong Yan

**Affiliations:** 1 State Key Laboratory for Diagnosis and Treatment of Infectious Diseases, Collaborative Innovation Center for Diagnosis and Treatment of Infectious Diseases, National Clinical Research Center for Infectious Diseases, National Medical Center for Infectious Diseases, The First Affiliated Hospital, Zhejiang University School of Medicine, 79 Qingchun Road, Hangzhou, 310003 China; 2 Department of Laboratory Medicine, College of Medicine, The First Affiliated Hospital, Zhejiang University, Hangzhou, 310003 China

**Keywords:** Hepatitis B, Acute-on-chronic liver failure, Artificial liver support system, Mass cytometry, Immunophenotype

## Abstract

**Background and aim:**

Liver failure, which is predominantly caused by hepatitis B (HBV) can be improved by an artificial liver support system (ALSS). This study investigated the phenotypic heterogeneity of immunocytes in patients with HBV-related acute-on-chronic liver failure (HBV-ACLF) before and after ALSS therapy.

**Methods:**

A total of 22 patients with HBV-ACLF who received ALSS therapy were included in the study. Patients with Grade I according to the ACLF Research Consortium score were considered to have improved. Demographic and laboratory data were collected and analyzed during hospitalization. Immunological features of peripheral blood in the patients before and after ALSS were detected by mass cytometry analyses.

**Results:**

In total, 12 patients improved and 10 patients did not. According to the immunological features data after ALSS, the proportion of circulating monocytes was significantly higher in non-improved patients, but there were fewer γδT cells compared with those in improved patients. Characterization of 37 cell clusters revealed that the frequency of effector CD8^+^ T (*P* = 0.003), CD4^+^ T_CM_ (*P* = 0.033), CD4^+^ T_EM_ (*P* = 0.039), and inhibitory natural killer (NK) cells (*P* = 0.029) decreased in HBV-ACLF patients after ALSS therapy. Sub group analyses after treatment showed that the improved patients had higher proportions of CD4^+^ T_CM_ (*P* = 0.010), CD4^+^ T_EM_ (*P* = 0.021), and γδT cells (*P* = 0.003) and a lower proportion of monocytes (*P* = 0.012) compared with the non-improved patients.

**Conclusions:**

Changes in effector CD8^+^ T cells, effector and memory CD4^+^ T cells, and inhibitory NK cells are associated with ALSS treatment of HBV-ACLF. Moreover, monocytes and γδT cells exhibited the main differences when patients obtained different prognoses. The phenotypic heterogeneity of lymphocytes and monocytes may contribute to the prognosis of ALSS and future immunotherapy strategies.

**Supplementary Information:**

The online version contains supplementary material available at 10.1186/s12865-023-00579-8.

## Introduction

Hepatitis B virus (HBV) is a severe and prevalent health problem globally [1]. Approximately 5–10% of adults infected with HBV become chronically infected, leading to chronic hepatitis, cirrhosis, hepatocellular carcinoma, and liver failure [2]. Liver failure, a syndrome of severe injury to liver cells, can manifest as acute liver failure, acute-on-chronic liver failure (ACLF), and end-stage liver disease [3, 4]. ACLF refers to the clinical syndrome of acute decompensated liver function in a short time based on chronic liver disease with high mortality [4–6].

Although the pathogenesis of ACLF remains unclear, the systemic inflammatory response is considered to play a key role in the process [6]. Dysfunction of the innate immune system is part of the immunopathology of ACLF and includes monocytes, neutrophils, dendritic cells, and macrophages [7]. Monocytes and macrophages are associated with the pathogenesis of ACLF [8, 9]. Bernsmeier et al. found that patients with ACLF had an increased number of monocytes and macrophages expressing tyrosine kinase receptors, which inhibited innate immune responses and promoted liver inflammation [10]. Recent studies reported significantly increased numbers of neutrophils in patients with ACLF, which was an independent risk factor for 28-day mortality, and a defect in the production of superoxide anion in neutrophils may lead to immunosuppression in ACLF [11–13]. Weiss et al. found a decreased proportion of memory lymphocytes and natural killer (NK) cells in the blood immune cell profile of patients with ACLF, and the selective depletion of these cells was an important factor in systemic immunosuppression [11].

Artificial liver support systems (ALSS) are an effective alternative to liver transplant for patients with liver failure, reducing short-term mortality in these patients [14]. In the past 30 years, ALSS, such as plasma exchange (PE) and molecular adsorbent recirculating system, have been used to detoxify the blood and improve liver function [15, 16]. Larsen et al. found that PE improved the prognosis of ACLF by removing plasma factor, cytokines, and immune mediators to reduce systemic inflammatory response syndrome [17]. A study by Qin and colleague demonstrated the efficacy and safety of PE-centric ALSS in supporting liver function and prolonging patient survival [15]. The Asian Pacific association for the study of the liver (APASL) recommended artificial liver treatment for ACLF (1, C) [4].

However, not all patients with liver failure benefit from ALSS treatment. Studies have shown that some biomarkers, cell subsets, and cytokines were helpful to judge the prognosis of ACLF. Liu et al. developed a generalized estimation equation that employs MELD score, creatinine, aspartate aminotransferase, and other biomarkers to forecast the outcome of ALSS in the patients with HBV-ACLF [18]. CD4^+^ CD25^+^ regulatory T cells levels to positively correlate with poor outcomes in HBV-ACLF, providing insight into short-term prognosis [19]. Li et al. reported that the reduction of NK cells was associated with poor prognosis of HBV-ACLF [20]. However, continuously elevated circulating Th22 cells and IL-22 were inversely correlated with the prognosis of HBV-ACLF [21]. In addition, various cytokines in the peripheral blood of patients with ACLF were elevated, especially IL-6 and IL-8, which were directly related to the occurrence and severity of ACLF [22–24]. Our previous studies have shown that the decrease of IL-28A level was an effective predictor of ALSS in the treatment of HBV-ACLF [25].

This study aimed to investigate the role of innate and adaptive immunity in liver injury following ALSS treatment by analyzing the immune characteristics of peripheral blood in patients with HBV-ACLF before and after ALSS. In addition, the study explored potential immunological mechanisms of different outcomes after ALSS therapy to assess the prognosis of ALSS and provide new insights and ideas for developing immunotherapy strategies for ACLF in the future.

## Patients and methods

### Study design

Patients with HBV-ACLF were included in the study in The First Affiliated Hospital, Zhejiang University School of Medicine from January 2020 to January 2021. The diagnosis and treatment of patients with ACLF was in accordance with the Acute-on-chronic liver failure: consensus recommendations of the Asian Pacific Association for the Study of the Liver (APASL) [4]. Data on clinical features and outcomes of patients were collected. Subsequently, PBMCs from all patients were analyzed by mass cytometry to identify potential differences in immunocytes. This study was conducted in compliance with the Declaration of Helsinki and was approved by the Ethical Committee of the First Affiliated Hospital of Zhejiang University of Medicine (No. 2017–674). All methods were carried out in accordance with relevant guidelines and regulations and informed consent was obtained from all subjects and their legal guardians.

### Patients

The study recruited hospitalized patients with clinical diagnosis of ACLF (as defined by the APASL [4]). The inclusion criteria were as follows: 1) early diagnosis of chronic liver disease and with hepatitis B surface antigen positive for 6 months; 2) ascites or hepatic encephalopathy complicated within 4 weeks; 3) serum bilirubin ≥ 5 mg/dL (85 µmol/L) and international normalized ratio ≥ 1.5. The 22 patients after applying exclusion criteria (Fig S1) were finally enrolled in the study and gave their informed consent prior to participation. Following three ALSS treatments (PE regimen) within six days, patients with Grade I (Table 1) were categorized as the improved group according to the ACLF Research Consortium score [4], while the other patients formed the non-improved group. In this study, the improvement of HBV-ACLF patients refers to the temporary normalization of liver failure markers and alleviation of clinical symptoms. The baseline characteristics recording demographic and laboratory data were collected during the hospitalization (Table 2).

**Table 1:** ACLF Research Consortium score and ACLF grade [4]

**AARC score**					
Points	Total Bilirubin (mg/dl)	HE Grade	INR	Lactate (mmol/l)	Creatinine (mg/dl)
** 1**	< 15	0	< 1.8	< 1.5	< 0.7
** 2**	15–25	I–II	1.8–2.5	1.5–2.5	0.7–1.5
** 3**	> 25	III–IV	> 2.50	> 2.5	> 1.5
**ACLF grade**					
Grade					Score
** I**					5–7
** II**					8–10
** III**					11–15

*ACLF* Acute-on-chronic liver failure, *INR* International normalized ratio

**Table 2:** Clinical characteristics of improved and non-improved groups of the patients with HBV-ACLF before and after the third treatment of ALSS

Characteristics	Improved-before (*n* = 12)	Non-improved-before (*n* = 10)	Improved-After (*n* = 12)	Non-improved-After (*n* = 10)	*P*-before	*P*-after
Demographics						
Age (years)	45.42 ± 15.12	46.7 ± 13.71	45.42 ± 15.12	46.7 ± 13.71	0.838	**-**
Sex (male/female)	10/2	8/2	10/2	8/2	0.840	**-**
Laboratory data						
HB (g/L)	118.00 ± 21.17	125.50 ± 16.27	113.17 ± 23.51	111.60 ± 10.87	0.370	0.772
PLT (10^9^/L)	83.08 ± 29.54	116.40 ± 45.45	70.08 ± 30.20	66.20 ± 23.42	0.051	1.000
TP (g/L)	55.21 ± 5.90	55.81 ± 5.11	54.83 ± 5.25	53.80 ± 5.03	0.806	0.539
ALB (g/L)	29.63 ± 2.75	31.25 ± 2.68	31.98 ± 4.69	32.43 ± 2.95	0.180	0.497
ALT (U/L)	154.28 ± 182.54	475.80 ± 383.12	58.17 ± 31.46	67.00 ± 39.31	0.018	0.674
AST (U/L)	89.25 ± 44.70	198 ± 149.12	51.67 ± 24.91	56.20 ± 35.97	0.073	0.974
TB (μmol/L)	355.75 ± 105.69	335.28 ± 103.54	167.75 ± 74.70	282.95 ± 106.44	0.653	**0.003**
DB (μmol/L)	249.63 ± 88.34	242.00 ± 88.63	131.55 ± 54.11	184.10 ± 53.29	0.843	**0.025**
Cr (μmol/L)	65.75 ± 22.76	63.70 ± 21.41	61.58 ± 15.48	59.90 ± 17.02	0.831	0.628
GFR (mL/min)	109.00 ± 22.77	108.43 ± 18.72	113.07 ± 16.90	111.28 ± 18.84	0.950	0.923
UA (μmol/L)	143.75 ± 70.90	110.30 ± 40.27	133.25 ± 35.81	116.00 ± 43.83	0.201	0.203
INR	1.89 ± 0.33	2.59 ± 0.66	1.70 ± 0.27	2.31 ± 0.63	**0.010**	**0.011**
PT (s)	20.76 ± 3.21	27.65 ± 7.36	19.18 ± 2.96	25.59 ± 7.08	**0.018**	**0.021**
AFP (ng/mL)	228.45 ± 224.14	210.60 ± 318.02	-	-	0.881	**-**
LA (mmol/l)	2.52 ± 0.47	3.29 ± 0.88	1.6 ± 0.22	2.74 ± 0.55	**0.028**	**< 0.001**
Score						
MELD	25.08 ± 2.90	28.80 ± 2.89	20.75 ± 2.95	26.00 ± 3.97	**0.007**	**0.003**
AARC	8.83 ± 1.26	10.10 ± 1.28	6.42 ± 0.66	9.80 ± 2.09	**0.032**	**< 0.001**
HBV-DNA	1.50*10^7^ ± 1.32*10^7^	3.03*10^7^ ± 1.72*10^7^	6.78*10^6^ ± 6.48*10^6^	2.56*10^7^ ± 1.64*10^7^	0.485	0.283
ACLF grade						
I/II/III	1/10/1	0/7/3	12/0/0	0/6/4	0.306	**< 0.001**

Data are shown as means ± SDs or number of patients (percentages)

*MELD* Model for End-Stage Liver Disease, *HBV-ACLF* Hepatitis B liver failure-related acute-on-chronic liver failure, *Sex* Sexuality, *HB* Hemoglobin, *PLT* Platelet, *TP* Total protein, *ALB* Albumin, *ALT* Alanine aminotransferase, *AST* Aspartate aminotransferase, *TB* Total bilirubin, *DB* Direct bilirubin, *Cr* Creatinine, *GFR* Glomerular filtration rate, *UA* Uric acid, *INR* International normalized ratio, *PT* Prothrombin time, *AFP* Alpha-fetoprotein, *LA* Lactic acid, *AARC* ACLF Research Consortium score

*Multiply

### Isolation of peripheral blood mononuclear cells (PBMCs)

Peripheral blood samples were collected from the median cubital vein of patients before and after the third treatment of ALSS, and heparin was added to the samples. PBMCs were extracted by Ficoll-Paque PLUS (17–1440-02, GE Healthcare, USA). Briefly, the blood was mixed with an equal volume of phosphate-buffered saline (PBS) and was layered on top of Ficoll without mixing (Ficoll: blood with PBS = 1:2) in a tube. The samples were centrifuged at 1500 r.p.m. for 10 min at 20 °C. PBMCs were collected from the interface between the plasma and Ficoll layer, washed twice with FACS Buffer (1.25% bovine serum albumin in PBS; BD Bioscience, USA), and subsequently centrifuged at 1500 r.p.m. for 5 min at 20 °C. PMBCs were resuspended with FACS Buffer, and the cell number and viability were calculated (Table S1). Frozen solution (90% FBS, 10% DMSO) was added to PBMCs, and the cell concentration was adjusted to 5*10^6^/mL, and then stored in liquid nitrogen. Samples were marked with unique bar-codes to decrease the batch effect and were sent to the laboratory for inspection on the same day.

### Mass cytometry (CyTOF) analyses

All PBMCs were stained for mass cytometry analyses, and the data were obtained by PLTTech Inc. (Hangzhou, China) using a CyTOF 2 Helios system (Fluidigm, South San Francisco, CA, USA). The frozen PBMCs were thawed in a 37 °C thermostatic water bath and then centrifuged at 1500 r.p.m. for 5 min. Following that, FASC Buffer was added to resuspend the cells, and the number and viability of cells were calculated (Table S1). Each sample was washed with 1 × PBS and then stained with 100 μL of 250 nM cisplatin on ice for 5 min. Following that, FACS Buffer was added at 4 °C and washed twice. Next, 50 μL Fc receptor blocking solution (Fluidigm) was added to the sample and blocked on ice for more than 20 min. Subsequently, 50 μL of the extracellular antibody mixture (Table S2) was added and stained on ice for 30 min. The cells were then washed twice using the same method. To fix the sample, a fixative solution (Fix and Perm buffer containing 191/193 Ir, Fluidigm) was added and the sample was fixed overnight at 4 °C. The following day, the cells were further fixed with Foxp3 nuclear staining reagent (Fluidigm) at room temperature for 30 min and washed twice with Perm buffer (eBioscience). Next, the intracellular antibody (Table S2) was added to the cells and stained on ice for 30 min. The cells were washed with FACS Buffer and resuspended in deionized water. Finally, the samples were counted, and the signal was detected using CyTOF. The types of immune cells were clustered by X-shift [26] clustering analysis and visualized by t-distributed stochastic neighbor embedding (tSNE) dimension reduction.

### Statistical analysis

Data were analyzed using SPSS v26.0 (IBM SPSS Inc.) software. In univariate statistical analyses, the results were presented as means ± SD. Significant differences between the two paired groups were determined by the paired *t*-test, and comparison between groups used one-sample *t*-test or Mann–Whitney U test. Enumeration data were expressed as percentages using the χ2 test. All data were analyzed using a two-sided test, and *P*-values < 0.05 were considered statistically significant.

## Results

### Characterization of peripheral blood immune cells

**Fig. 1:**
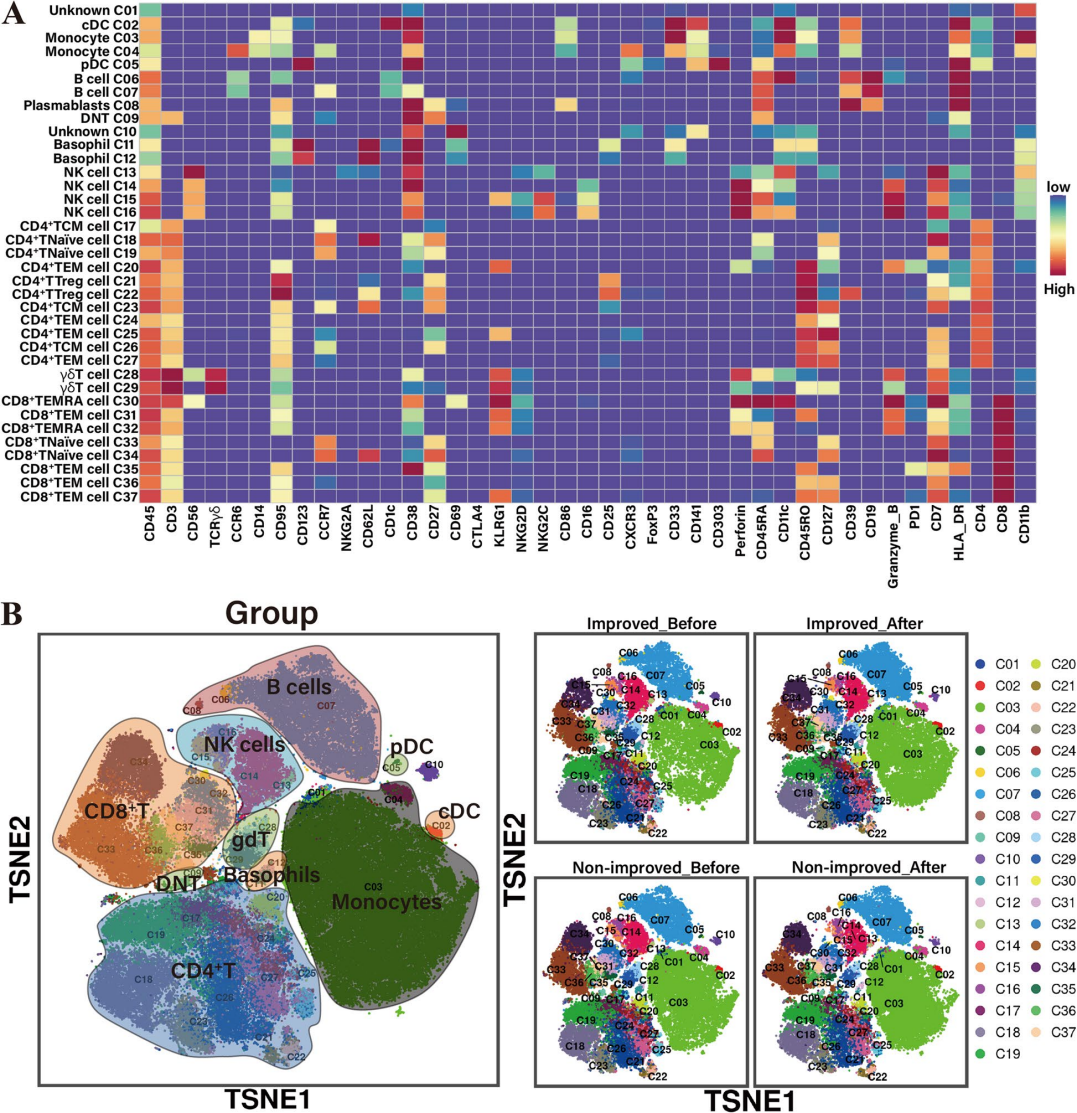
Peripheral blood immune cell cluster sorting according to marker expression levels. **A** Heatmap of normalized immune cell marker expressing in 37 immune cell clusters. **B** T-SNE map was colored by clusters, displaying 40 000 cells from PBMC analyze with immune cell clusters in all samples. 37 immune cell clusters were divided into 9 cell lineages. T-SNE maps displaying 40 000 cells from PBMC analyzed with immune cell clusters in two groups before and after ALSS treatment. t-SNE: t-distributed stochastic neighbor embedding; PBMC: peripheral blood mononuclear cells; ALSS: artificial liver support system

A total of 22 patients with HBV-ACLF who received ALSS treatment were enrolled in the study at The First Affiliated Hospital, Zhejiang University School of Medicine. According to the outcomes of ALSS treatment, 12 of the 22 patients improved and 10 patients did not recover. All cells were divided into different phenotypes based on marker expression and the clustering algorithm (Fig. 1A). CD45^+^ immune cells are classified into 37 cell subsets (C01–37) and the differences in these subsets between the improved and non-improved groups, and before and after ALSS therapy, are shown in Fig. 1B. The classification of all CD45^+^ immune cells, including the expression of key marker molecules, is provided in Table S3. The 37 cell clusters belong to nine lineages: CD4^+^ T cells, CD8^+^ T cells, γδT cells, DNT cells, NK cells, dendritic cells, basophils, monocytes, and B cells (Fig. 1B). The immune cell lineages in the PBMCs of the two groups were analyzed by the CyTOF. The proportion of monocytes and γδT cells was significantly different in the total lineage. Furthermore, there were some significant differences between the two groups in subgroups, particularly in CD8^+^ T cells, CD4^+^ T cells, NK cells, γδT cells, and monocytes.

### CD8^+^ T-cell clusters in HBV-ACLF

**Fig. 2:**
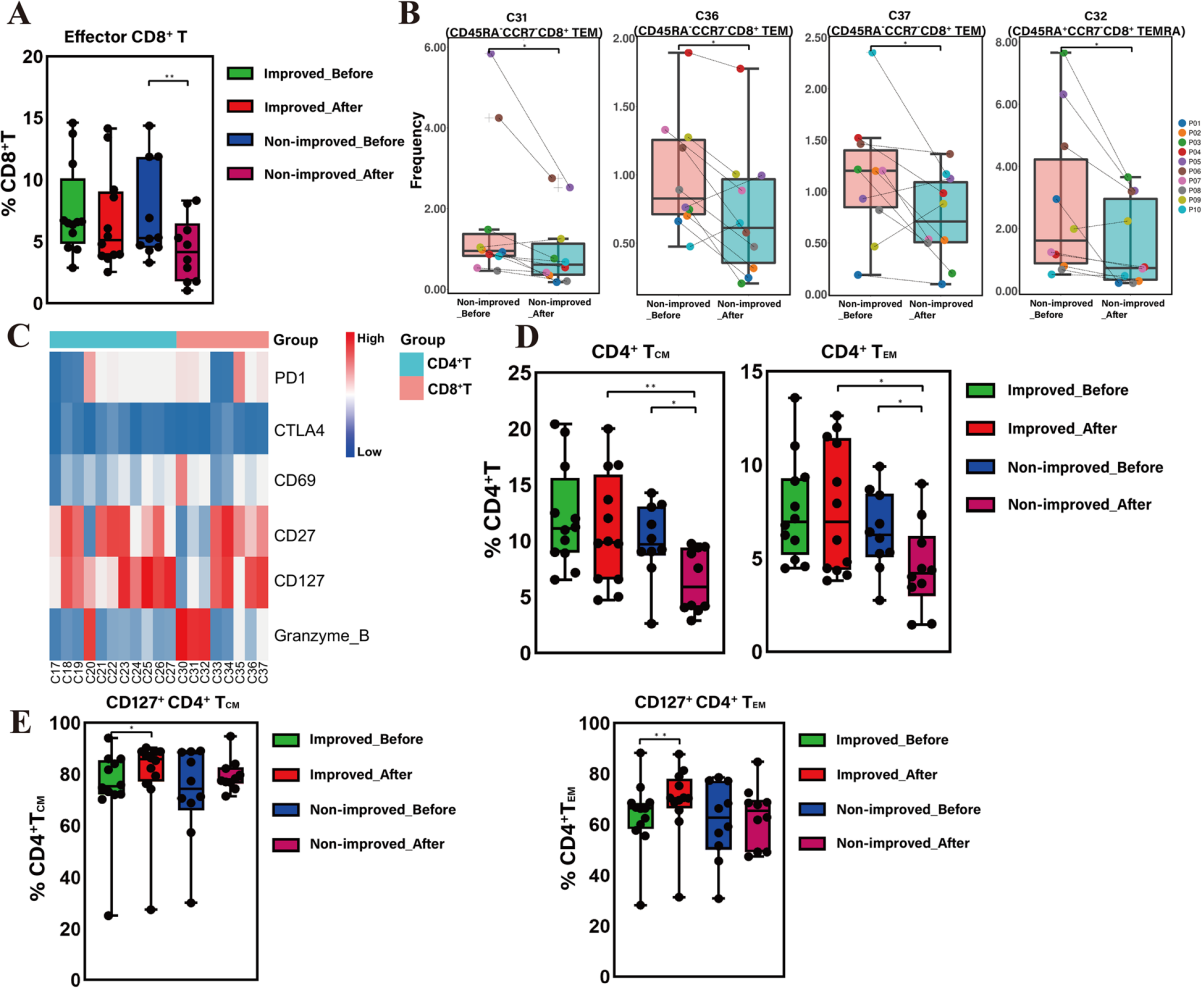
The frequency of CD8^+^ T-cell and CD4^+^ T-cell subsets between improved and non-improved patients before and after ALSS therapy. **A** The frequency of effector CD8^+^ T in non-improved patients was significantly lower after ALSS therapy. **B** The frequency of CD8^+^ T_EM_ (C31, C36, C37) and CD8^+^ T_EMRA_ (C32) in non-improved patients was significantly lower after ALSS therapy. **C** Heatmap of PD1, PD1, CTLA4, CD69, CD27, CD127, and Granulozyme B expression in CD4T^+^ and CD8T^+^ cell clusters. **D** The frequency of CD4^+^ T_CM_ and CD4^+^ T_EM_ in non-improved patients decreased significantly after ALSS and was lower than in improved patients. **E** The frequency of CD127^+^ T_CM_ and CD4^+^ T_EM_ in improved patients was higher than that before ALSS therapy. *p* < 0.05 was considered statistically significant, *p < 0.05. **p < 0.01. ALSS: artificial liver support system; EM: effector memory; EMRA: terminally differentiated effector memory; CM: central memory

The eight CD8^+^ T cell clusters (C30–C37) were divided into two subtypes—effector CD8^+^ T (C30–C32, C35–C37) and naïve CD8^+^ T (C33, C34). Comparison of the CD8^+^ T cell subtypes between the two groups before and after treatment revealed that the frequency of effector CD8^+^ T cells in non-improved patients decreased significantly after ALSS (*P* = 0.003) (Fig. 2A). Similar manifestations were observed in improved patients although the difference was not statistically significant by paired *t*-test (Fig. 2A). However, there was no significant difference in naïve CD8^+^ T cells between the two groups before and after treatment (Fig. S2A).

Six clusters of effector CD8^+^ T cells were further analyzed in non-improved patients before and after ALSS treatment. Effector memory cells (T_EM_) (C31, C36, and C37; *P*_*C31*_ = 0.044, *P*_*C36*_ = 0.013, *P*_*C37*_ = 0.037) and terminally differentiated effector memory cells (T_EMRA_) (C32, *P* = 0.021) were the main CD8^+^ T phenotypes that decreased after ALSS treatment (Fig. 2B). Figure 2C shows the expression of PD1, CTLA4, CD69, CD27, CD127, and granzyme B in CD8^+^ T cells. Cluster analysis indicated that there were no statistically significant differences in the expression of these proteins on the surface of effector and naïve CD8^+^ T cells before and after ALSS (Fig. S2B and C).

### CD4^+^ T cell clusters in HBV-ACLF

Eleven clusters of CD4^+^ T cells were divided into four subtypes: central memory CD4^+^ T cells (C17, C23, C26, CD4^+^ T_CM_), effector memory CD4^+^ T cells (C20, C24, C25, C27, CD4^+^ T_EM_), Treg CD4^+^ T cells (C21, C22), and naïve CD4^+^ T cells (C18, C19). The proportion of CD4^+^ T_CM_ (*P* = 0.028) and CD4^+^ T_EM_ (*P* = 0.017) cells in non-improved patients decreased significantly after ALSS therapy, while there was no significant difference in these subsets in improved patients before and after treatment (Fig. 2D). After ALSS, the frequency of CD4^+^ T_CM_ (*P* = 0.010) and CD4^+^ T_EM_ (*P* = 0.021) in improved patients was significantly higher compared with those in non-improved patients, but this difference between the two groups was not observed before treatment (Fig. 2D).

In addition, the expression of PD1, CTLA4, CD69, CD27, CD127, and granzyme B was examined in CD4^+^ T_CM_ and CD4^+^ T_EM_ cells. In improved patients, the proportions of CD4^+^ T_CM_ (*P* = 0.021) and CD4^+^ T_EM_ (*P* = 0.003) cells expressing CD127 were significantly increased after ALSS treatment (Fig. 2E).

### NK cell clusters in HBV-ACLF

**Fig. 3:**
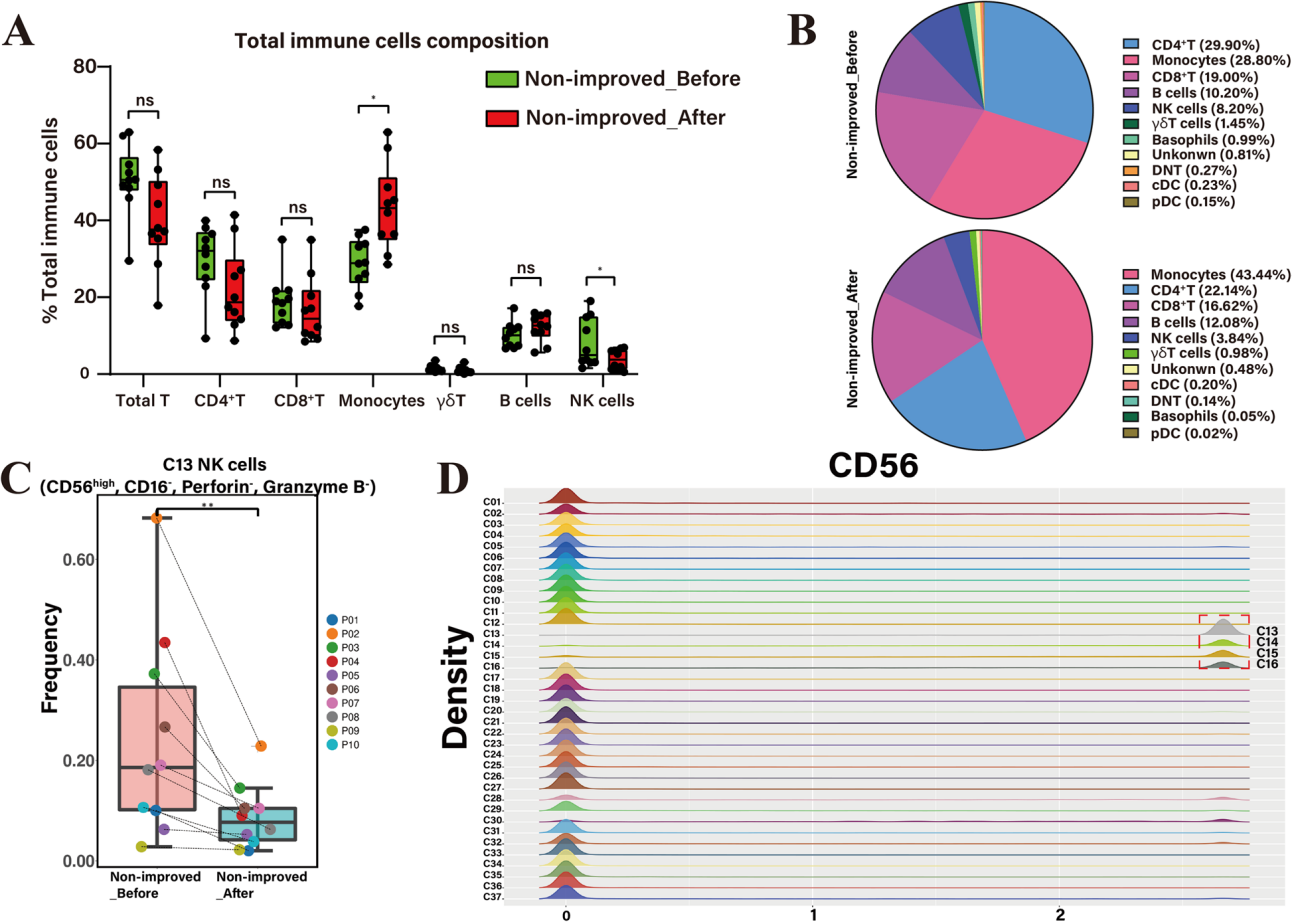
Comparison of NK cells and clusters in non-improved patients before and after ALSS therapy. **A** Boxplots displaying the variation of immune cells in non-improved patients at the total immune cells level. **B** Pie charts displaying the frequency of immune cell subsets in the PBMC before and after ALSS therapy. **C** The frequency of C13 (CD56^high^ NK cell) significantly reduced in non-improved patients after ALSS. **D** The expression of CD56 in 37 clusters. C13–16 were marked with red dotted box. *p* < 0.05 was considered statistically significant, *p < 0.05. **p < 0.01. NK: natural kill; ALSS: artificial liver support system; PBMC: peripheral blood mononuclear cells

There were significant differences in the proportion of NK cells before and after treatment in non-improved patients (*P* = 0.029) (Fig. 3A), with the proportion of NK cells reducing from 8.20% to 3.84% (Fig. 3B). Further analysis of the cell clusters revealed that C13 (CD56^high^, CD16^–^, Perforin^–^, Granzyme B^–^) (*P* = 0.0083) was the predominant reduced subset of NK cells (Fig. 3C). There were reductions in the other three clusters–C14 (CD56^+^, Perforin^+^, Granzyme B^+^), C15 (CD56^+^, KLRG1^+^, NKG2C^+^, Perforin^+^, Granzyme B^+^, CD11c^–^), and C16 (CD56^+^, NKG2C^+^, Perforin^+^, Granzyme B^+^) after ALSS in the non-improved group (Fig. S2D), but no significant difference was detected.

All four clusters of NK cells expressed CD56, but the density of CD56 surface expression was different (Fig. 3D). NK cells have been classified as CD56^high^ NK cells according to the high expression titer of CD56 [27]. In general, a decrease in NK cells, especially CD56^high^ NK cells, was detected after ALSS in patients with HBV-ACLF.

### γδT cell clusters in HBV-ACLF

**Fig. 4:**
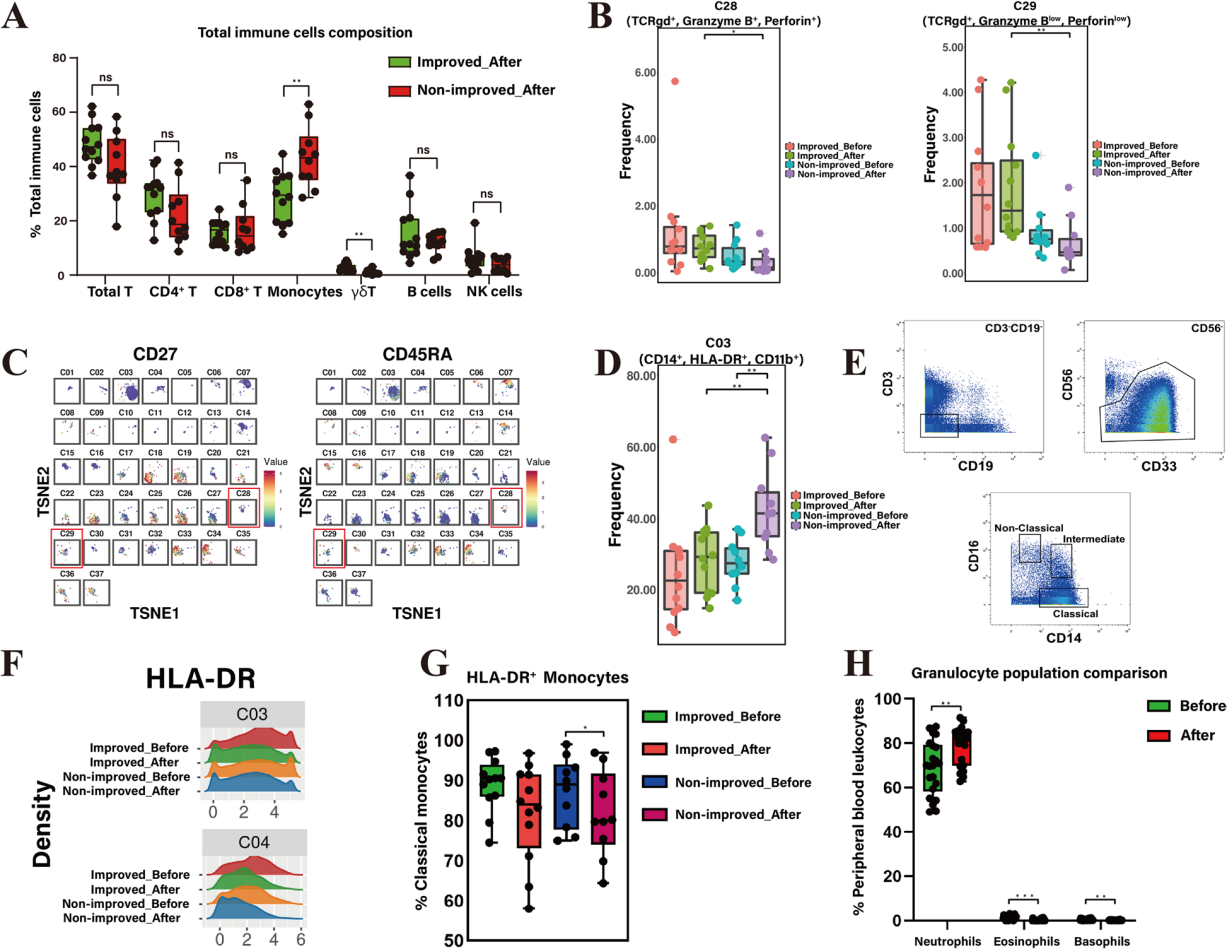
Comparison of γδT cells, monocytes, granulocytes, and clusters between improved and non-improved patients after ALSS therapy. **A** Boxplots displaying the difference between immune cell subsets of two groups in total immune cell level after ALSS therapy. **B** The frequency of γδT cell clusters (C28 and C29) of improved patients was significantly higher compared to non-improved patients. **C** T-SNE maps displaying the expression of CD27 and CD45RA in 37 clusters. C28 and C29 were marked with red boxes. **D** The frequency of monocytes (C03) in non-improved patients with ALSS was significantly higher than that before treatment, and was significantly higher compared to improved patients. **E** Sequential gating strategy for identification of classical monocytes (CD14^++^ CD16^–^), intermediate monocytes (CD14^++^ CD16^+^), and non-classical monocytes (CD14^+^ CD16^++^) using immunophenotyping followed by CyTOF. **F** The expression of HLA-DR between improved and non-improved patients before and after ALSS therapy. **G** The frequency of HLA-DR expression in classical monocytes in the improved and non-improved groups before and after ALSS. **H** The frequency of eosinophils and basophils in all samples decreased but neutrophils increased after ALSS.* p* < 0.05 was considered statistically significant, *p < 0.05. **p < 0.01. ***p < 0.001 ALSS: artificial liver support system; t-SNE: t-distributed stochastic neighbor embedding; CyTOF: mass cytometry

γδT cells (*P* = 0.003) were one of the predominant different immune cells between the improved and non-improved groups after ALSS treatment (Fig. 4A). However, there was no significant difference in immune cells lineage between two groups before treatment (Fig. S3A). C28 (TCRγδ^+^, Granzyme B^+^, Perforin^+^) and C29 (TCRγδ^+^, Granzyme B^low^, Perforin^low^) were two subsets of γδT cells. Following ALSS therapy, C28 (*P* = 0.013) and C29 (*P* = 0.008) were present at a higher frequency in improved patients compared with those in non-improved patients (Fig. 4B). However, there was no significant difference between the two groups prior to treatment (Fig. 4B).

In addition, the expression of cell surface proteins on C28 and C29 was examined. CD27 expression was not detected in C28 and C29 subsets, while CD45RA expression was detected in C28 but not in C29 (Fig. 4C). C28 (CD27^–^ CD45RA^+^) belonged to the terminally differentiated phenotype and C29 (CD27^–^ CD45RA^–^) belonged to the effector memory phenotype. In conclusion, the frequencies of terminally differentiated γδT cells and effector memory γδT cells in improved patients were higher than those in non-improved patients after ALSS therapy.

### Monocytes in HBV-ACLF

After ALSS, the proportion of monocytes (*P* = 0.012) was significantly increased in non-improved patients (Fig. 3A), with the proportion increasing from 28.80% to 43.44% (Fig. 3B). A significant difference was also observed in monocytes (*P* = 0.004) between improved and non-improved patients after ALSS therapy (Fig. 4A). Further analysis showed that C3 (CD14^+^, HLA-DR^+^, CD11b^+^) was the main monocyte cluster that increased in non-improved patients after ALSS (Fig. 4D).

In addition, monocytes are further divided into three subtypes according to the expression of CD14 and CD16 (Fig. 4E); these subtypes are classical monocytes (CD14^++^ CD16^–^), intermediate monocytes (CD14^++^ CD16^+^), and non-classical monocytes (CD14^+^ CD16^++^) [28]. The proportion of monocyte subsets from the mean ± SD of all samples was 87.7% ± 1.8% for classical, 8.4% ± 1.4% for intermediate, and 4.2% ± 0.7% for non-classical. The proportion of these three subsets of monocytes before and after ALSS was analyzed, but no significant difference was observed (Fig. S3B). As shown in Fig. 4F, monocytes (C03, C04) also highly expressed HLA-DR. Further comparison of the expression of HLA-DR in the three subsets between the two groups showed that the frequency of classical monocytes with high expression of HLA-DR in the non-improved patients was significantly reduced after treatment (*P* = 0.025) (Fig. 4G).

### Granulocytic cells in HBV-ACLF

Laboratory examination data of all patients during hospitalization were collected, and the granulocyte population of complete blood count was analyzed. The proportion of neutrophils in the improved and non-improved groups increased significantly after ALSS treatment (*P* = 0.002), while eosinophils (*P* < 0.001) and basophils (*P* = 0.005) decreased significantly (Fig. 4H). However, there was no significant difference in the proportion of neutrophils, eosinophils and basophils in peripheral blood between the improved group and the non-improved group after ALSS (Fig. S3c).

### HBV-DNA in HBV-ACLF

In addition, the viral load data of all patients with HBV-ACLF before and after ALSS were collected in Table 2. The results indicated that ALSS treatment had the potential to ameliorate viremia in HBV-ACLF cases. The frequency of immune cells was associated with viremia levels. Study have demonstrated a positive correlation between T-cell activation levels and HIV-RNA levels [29]. However, in this study, no significant difference in viral load was observed between the improved and non-improved groups before (*P* = 0.485) and after (*P* = 0.283) ALSS treatment.

##  Discussion

In this study, immunological analysis of patients with HBV-ACLF was performed after artificial liver treatment. Patients with HBV-ACLF who failed to recover after ALSS treatment had distinct populations of certain immune cells, such as CD8^+^ T_EM_, CD8^+^ T_EMRA_, CD4^+^ T_CM_, and CD4^+^ T_EM_. Moreover, the improved patients had a higher frequency of γδT cells and a lower frequency of monocytes compared with the non-improved patients after therapy. These discoveries show the difference in immune background between different outcomes in HBV-ACLF after ALSS.

The frequency of CD8^+^ T_EM_ and T_EMRA_ subsets in PBMCs of non-improved patients were significantly reduced after ALSS treatment. CD8^+^ T_EM_ is the main subset of circulating memory CD8^+^ T cells and has cytotoxicity potential [30, 31]. CD8^+^ T_EMRA_ is the terminally differentiated cell with high cytotoxicity, transformed from CD8^+^ T_EM_ by proliferation and differentiation [32]. The control of liver disease was previously reported to be closely related to recovery of the HBV-specific T cell response [33]. Moreover, another study demonstrated that in acute hepatitis B, influenza, and other viral diseases, the virus was gradually cleared as the level of CD8^+^ T_EM_ increased [34]. Ma et al. suggested that a significant increase in the CD8^+^ T_EMRA_ cell subset contributes to HBV clearance [35]. In addition, this study found that the frequency of CD4^+^ T_CM_ and T_EM_ decreased significantly in the non-improved group after ALSS. CD4^+^ T cells are critical in the development of CD8^+^ T cells and promote the immune response of effector and memory CD8^+^ T cells [36, 37]. Shi et al. found that HBV-specific CD4^+^ T_CM_ and T_EM_ increased in patients with HBV during immune activation [38]. Furthermore, in this study, the proportion of CD4T^+^ T_CM_ and CD4^+^ T_EM_ cells expressing CD127 was significantly increased in improved patients after therapy [39]. CD127 (IL-7Rα) is involved in the regulation of immune homeostasis. The binding of CD127 to its ligand IL-7 leads to the up-regulation of anti-apoptotic molecules and the enhancement of TCR-mediated signal transduction, resulting in the proliferation of activated T cells [40]. Similar findings were observed in our study. The decrease of effector and memory T-lymphocytes in non-improved patients after ALSS may be due to the disease progression. Further comparison between the improved and non-improved groups showed that CD8^+^ T_EM_, CD8^+^ T_EMRA_, CD4^+^ T_CM_, and CD4^+^ T_EM_ accounted for a lower proportion of PBMCs in non-improved patients, indicating that uncured patients had immune tolerance. These results explain the reasons for different outcomes after ALSS to some extent. ALSS based on plasma exchange therapy offers valuable benefits in correcting coagulation disorders, eliminating liver toxins, and enhancing liver function among individuals suffering from liver failure [41]. The application of plasma exchange contributes to a favorable prognosis for ALF by mitigating the activation of the innate immune system [17]. In addition, ALSS treatment has shown significant efficacy in ameliorating cytokine storms and inflammation in patients diagnosed with coronavirus disease 2019 [42]. In the study, the proportion of CD4^+^ T and CD8^+^ T memory cells in HBV-ACLF patients decreased after ALSS, suggesting that ALSS therapy may improve liver inflammation and inhibit the activation of the immune system. Our findings supplement the research on T cells in the immunity of hepatitis B.

NK cells, especially CD56^high^ NK cells, decreased in patients with HBV-ACLF after ALSS. NK cells play a pivotal role in the pathogenesis of HBV as a part of innate immunity [43, 44]. CD56^high^ NK cells are inhibitory NK cells and specifically produce inflammatory cytokines, such as interferons, which are instrumental in clearing infectious pathogens [45, 46]. Boni et al. found that the inflammatory phenotype of NK cells in patients with hepatitis B decreased after viremia suppression by nucleotide analogue therapy [47]. In our study, a reduction in the number of inhibitory NK cells indicated an improvement in viremia of HBV-ACLF after ALSS, demonstrating that artificial liver therapy has the potential to improve viral load.

The frequency of γδT cells was higher in improved patients compared with that in non-improved patients after ALSS. γδT cells are unique T lymphocytes with the function of connecting innate immunity and acquired immunity. The function of the γδT cell in HBV infection depends on the stage of the disease. Chen et al. demonstrated that peripheral and intrahepatic γδT cells in patients with chronic hepatitis B (CHB) changed from tolerance to activation as the disease progressed, and the frequency of γδT cells decreased [48]. Wang et al. considered that γδT cells in patients with CHB shield the host from HBV infection [49]. In acute hepatitis, the proportion of γδT cells in peripheral blood was adversely linked with disease severity [50]. Moreover, there is evidence that γδT cells could be used to improve liver injury in HBV carriers [51]. However, another study showed that γδT cells cause liver injury in patients with HBV-ACLF by producing inflammatory cytokines [52]. In addition, γδT cells of the CHB mouse model mobilized myeloid-derived suppressor cells for infiltration into the liver to mediate CD8^+^ T cell failure and immune tolerance [53]. In terms of liver regeneration, Rao et al. suggested that γδT cells induced a pro-regeneration phenotype of inflammatory hepatocytes and promoted liver regeneration [54]. There is also evidence suggesting that the mechanism of microbiota promoting liver regeneration might be closely related to γδT cells [55]. ALSS, originating from the concept of liver regeneration, has the potential to prevent hepatocyte necrosis and stimulate hepatocyte regeneration [56, 57]. Several studies have shown that γδT cells have a protective effect by regulating the apoptosis of pathogenic T cells to manage inflammation and promote disease recovery [58, 59]. Collectively, part of these studies may help to explain why the frequency of γδT cells was higher in improved patients in our study. Moreover, C28 and C29 were the main subsets of γδT cells that changed in our study. C28 (T_EMRA_) and C29 (T_EM_) are activated effector γδT cells that express the granzyme B and perforin to kill target cells [60]. Therefore, the increase in γδT cells in improved patients likely had a protective effect. Our study expands the understanding of the role of γδT cells in patients with HBV and complements the immune mechanism of artificial liver therapy.

Findings from this study indicate that high levels of monocytes were related to the non-improved prognosis of HBV-ACLF patients treated with ALSS. Monocytes have the most number of categories in leukocytes, accounting for 2–8% of total leukocytes in the blood [61]. Previous studies showed that, following infection of humans with HBV, activated monocytes were released into the blood to contribute to the systemic inflammatory state by producing many pro-inflammatory cytokines, which progressively lead to hepatocyte injury [62]. In addition, injured hepatocytes further reacted against the immune system, leading to an immunodeficiency state [63]. In this study, the frequency of classical monocytes expressing HLA-DR in HBV-ACLF patients with poor prognosis (non-improved group) was reduced after ALSS treatment. HLA-DR expressed by monocytes can present antigens to T cells and activate adaptive immunity [64]. Some studies have suggested that low monocyte HLA-DR expression might indicate an immunosuppressive condition [65, 66]. Our results support prior findings and demonstrate that immunosuppression might be the reason why some patients with HBV-ACLF do not benefit from artificial liver therapy.

In our study, there was no significant difference in B lymphocytes between the groups (Figs. 3A and 4A). B cells express clonal diversity of cell surface immunoglobulin receptors that recognize specific antigen epitopes and play an important role in humoral immunity [67]. In the past 20 years, most of the studies on the pathogenesis of HBV have focused on T cell populations, overlooking the significance of B cells and humoral immune responses for an extended period [68]. A recent study has revealed that patients with HBV-ACLF exhibit elevated levels of circulating CD19^+^ B cells, as well as increased serum and liver IgG/M levels compared to individuals with CHB and healthy people [69]. In addition, Zhao et al. found that intrahepatic B cells in ACLF showed enhanced activation and altered effector functions [70]. However, Li et al. reported that there was no significant difference between ACLF and non-ACLF in CD19^+^ B cells, and no significant difference was noted between ACLF survivors and non-survivors as well [71]. ALSS includes plasma exchange therapy. Early studies have reported that plasma exchange has no significant effect on peripheral blood albumin and IgG levels, and no damage to humoral immunity [72]. Our study found similar results, but further research is necessary to elucidate the role of B cells and humoral immunity in HBV-ACLF.

In addition, the proportion of neutrophils in the peripheral blood of patients with HBV-ACLF increased after ALSS treatment, while that of eosinophils and basophils decreased. Neutrophils have been used to predict the development and progression of liver disease [73]. Li et al. developed a new predictive score for HBV-ACLF using six predictors including neutrophils [74]. Our data supplement the changes of peripheral blood granulocytes in HBV-ACLF patients after ALSS.

There are several limitations to the study. First, the study lacks separate medication and healthy controls, and the interference of drugs with the immune system is easily ignored. Second, the relatively small number of samples in the study may produce outcome bias. Third, the data on patients with HBV-ACLF were collected from one medical center, so this cohort may not be representative of the general population, some important immunological differences may be missing, and correlations between biomarkers and measures of liver function may have been overlooked. Fourth, no in vitro culture or animal experiments were performed to detect the function of the immune cells. In our future work, the cell clusters of interest will be selected to further explore the immune mechanism of HBV-ACLF in different outcomes after artificial liver treatment. In addition, large-sample multicenter studies are needed to consider the above factors and establish and validate whether monocytes and γδT cells can predict the outcome of patients with HBV-ACLF after ALSS.

##  Conclusion

In summary, our data reports some alterations in peripheral blood immune cells of patients with HBV-ACLF following ALSS treatment. Effector CD8^+^ T cells, effector and memory CD4^+^ T cells, and inhibitory NK cells exhibited differences after treatment. In particular, γδT cells, monocytes, and part of the T-lymphocytes clusters can explain the potential immune mechanisms of different treatment outcomes and provide evidence to support artificial liver treatment of HBV-ACLF. This finding supplies an immunological explanation for the divergent results of HBV-ACLF patients after ALSS therapy and highlights potential directions for HBV-ACLF immunotherapy.

### Supplementary Information

**Additional file 1:** **Table S1.** Cryopreservation and resuscitation of PBMCs. **Table S2.** CyTOF panel. **Table S3.** The classification of all CD45^+^ immune cells and the key marker expression. **Fig. S1.** Flow chart of inclusion of HBV-ACLF patients treated with ALSS. HBV-ACLF: hepatitis B virus-related acute-on-chronic liver failure; ALSS: artificial liver support system. **Fig. S2.** A. The proportion of naïve CD8^+^ T between improved and non-improved patients before and after ALSS therapy. B. The expression of PD1, CTLA4, CD69, CD27, CD127, and Granzyme B on effector CD8^+^ T cells in the two groups before and after treatment. C. The expression of PD1, CTLA4, CD69, CD27, CD127, and Granzyme B on naïve CD8^+^ T cells in the two groups before and after treatment. D. The frequency of C14, C15, and C16 (NK cells) clusters between improved and non-improved patients before and after ALSS therapy. NK: natural kill; ALSS: artificial liver support system. **Fig. S3.** A. Boxplots displaying the difference between immune cell subsets of two groups in total immune cell level before ALSS therapy. B. The proportion of classical, intermediate, and non-classical monocytes between improved and non-improved patients before and after ALSS therapy. C. The changes of granulocytes in peripheral blood of two groups before and after treatment. ALSS: artificial liver support system.

## Data Availability

The datasets used or analyzed during the current study are available from the corresponding author upon reasonable request.

## Abbreviations

HBV: Hepatitis B virus
ALSS: Artificial liver support system
HBV-ACLF: HBV-related acute-on-chronic liver failure
ACLF: Acute-on-chronic liver failure
NK: Natural killer
PE: Plasma exchange
ALF: Acute liver failure
APASL: Asian Pacific Association for the Study of the Liver
PBMC: Peripheral blood mononuclear cells
PBS: Phosphate-buffer saline
CyTOF: Mass cytometry
tSNE: T-distributed stochastic neighbor embedding
CHB: Chronic hepatitis B
