# Supplementary material for: Characteristics of circulating immune cells in HBV-related acute-on-chronic liver failure following artificial liver treatment
Source: BMC Immunol. 2023 Nov 25;24:47. doi: 10.1186/s12865-023-00579-8 (PMC10676598; doi:10.1186/s12865-023-00579-8)
Supplement: Supplementary file 1 — Additional file 1: Table S1. Cryopreservation and resuscitation of PBMCs. Table S2. CyTOF panel. Table S3. The classification of all CD45+ immune cells and the key marker expression. Fig. S1. Flow chart of inclusion of HBV-ACLF patients treated with ALSS. HBV-ACLF: hepatitis B virus-related acute-on-chronic liver failure; ALSS: artificial liver support system. Fig. S2. A. The proportion of naïve CD8+ T between improved and non-improved patients before and after ALSS therapy. B. The expression of PD1, CTLA4, CD69, CD27, CD127, and Granzyme B on effector CD8+ T cells in the two groups before and after treatment. C. The expression of PD1, CTLA4, CD69, CD27, CD127, and Granzyme B on naïve CD8+ T cells in the two groups before and after treatment. D. The frequency of C14, C15, and C16 (NK cells) clusters between improved and non-improved patients before and after ALSS therapy. NK: natural kill; ALSS: artificial liver support system. Fig. S3. A. Boxplots displaying the difference between immune cell subsets of two groups in total immune cell level before ALSS therapy. B. The proportion of classical, intermediate, and non-classical monocytes between improved and non-improved patients before and after ALSS therapy. C. The changes of granulocytes in peripheral blood of two groups before and after treatment. ALSS: artificial liver support system. [file 12865_2023_579_MOESM1_ESM.zip › Supplementary.DOCX]

Supplementary Material

**Characteristics of circulating immune cells in HBV-related acute-on-chronic liver failure following artificial liver treatment**

**Supplementary Figures and Tables**

**Supplementary tables:**

Table s1. Cryopreservation and resuscitation of PBMCs

|  | | | Before cryopreservation | | | Post-resuscitation | | |
| --- | --- | --- | --- | --- | --- | --- | --- | --- |
| Sample  number | viable cell count (10^6^) | survival rate (%) | | Number of frozen tubes | | Number of resuscitation tubes | viable cell count (10^6^) | survival rate (%) |
| P1 before | 5.65 | 96.58 | | | 2 | 1 | 0.98 | 76.27 |
| P2 before | 3.41 | 92.89 | | | 2 | 1 | 1.08 | 92.82 |
| P3 before | 8.00 | 94.39 | | | 2 | 1 | 2.61 | 94.30 |
| P4 before | 5.43 | 95.11 | | | 2 | 1 | 1.62 | 90.45 |
| P5 before | 18.03 | 95.66 | | | 4 | 1 | 2.39 | 81.32 |
| P6 before | 12.37 | 92.83 | | | 3 | 1 | 2.41 | 78.62 |
| P7 before | 8.63 | 91.48 | | | 2 | 1 | 2.11 | 77.31 |
| P8 before | 12.71 | 91.27 | | | 3 | 1 | 3.34 | 83.44 |
| P9 before | 14.16 | 96.72 | | | 3 | 1 | 2.53 | 86.50 |
| P10 before | 8.19 | 92.21 | | | 2 | 1 | 1.95 | 84.91 |
| P11 before | 7.28 | 84.48 | | | 2 | 1 | 1.21 | 79.53 |
| P12 before | 6.29 | 95.75 | | | 2 | 1 | 2.45 | 88.80 |
| P13 before | 10.68 | 90.34 | | | 2 | 1 | 1.80 | 64.00 |
| P14 before | 10.04 | 97.41 | | | 2 | 1 | 3.00 | 91.81 |
| P15 before | 3.63 | 86.84 | | | 2 | 2 | 1.94 | 84.31 |
| P16 before | 12.74 | 96.78 | | | 3 | 1 | 1.97 | 74.31 |
| P17 before | 10.15 | 96.04 | | | 2 | 1 | 2.60 | 70.78 |
| P18 before | 7.10 | 95.62 | | | 2 | 1 | 2.84 | 81.72 |
| P19 before | 15.21 | 99.15 | | | 3 | 1 | 3.33 | 92.34 |
| P20 before | 9.18 | 95.12 | | | 2 | 1 | 2.39 | 93.51 |
| P21 before | 22.90 | 96.57 | | | 4 | 1 | 3.12 | 75.39 |
| P22 before | 11.92 | 89.79 | | | 2 | 1 | 3.96 | 87.72 |
| P1 after | 10.96 | 95.18 | | | 2 | 1 | 1.44 | 59.69 |
| P2 after | 12.00 | 98.11 | | | 3 | 1 | 2.36 | 93.55 |
| P3 after | 11.77 | 95.95 | | | 2 | 1 | 3.33 | 86.59 |
| P4 after | 6.16 | 97.28 | | | 2 | 1 | 1.61 | 81.43 |
| P5 after | 27.76 | 97.45 | | | 5 | 1 | 2.99 | 80.77 |
| P6 after | 29.31 | 96.24 | | | 5 | 1 | 3.12 | 80.88 |
| P7 after | 5.13 | 96.57 | | | 2 | 2 | 1.58 | 60.40 |
| P8 after | 18.40 | 93.77 | | | 4 | 1 | 2.82 | 74.81 |
| P9 after | 7.88 | 98.83 | | | 2 | 1 | 1.67 | 82.53 |
| P10 after | 22.10 | 99.09 | | | 4 | 1 | 3.74 | 91.17 |
| P11 after | 11.6 | 98.13 | | | 3 | 1 | 2.40 | 74.18 |
| P12 after | 10.62 | 95.62 | | | 2 | 1 | 3.24 | 84.67 |
| P13 after | 15.71 | 91.64 | | | 3 | 1 | 2.44 | 74.00 |
| P14 after | 11.22 | 98.52 | | | 2 | 1 | 3.44 | 85.07 |
| P15 after | 25.08 | 99.09 | | | 5 | 1 | 1.59 | 65.86 |
| P16 after | 14.73 | 92.60 | | | 3 | 1 | 2.48 | 73.04 |
| P17 after | 11.99 | 98.06 | | | 3 | 1 | 2.22 | 82.19 |
| P18 after | 8.96 | 98.69 | | | 2 | 1 | 2.34 | 87.23 |
| P19 after | 25.22 | 96.81 | | | 5 | 1 | 2.67 | 75.87 |
| P20 after | 6.74 | 99.11 | | | 2 | 1 | 1.75 | 93.08 |
| P21 after | 7.18 | 99.31 | | | 2 | 1 | 1.77 | 90.12 |
| P22 after | 8.86 | 96.70 | | | 2 | 1 | 2.49 | 81.89 |

Table s2. CyTOF panel

| ID | Channel | Antibody | ID | Channel | Antibody |
| --- | --- | --- | --- | --- | --- |
| 1 | 89Y | CD45 | 22 | 159Tb | CD16 |
| 2 | 115In | CD3 | 23 | 160Gd | CD25 |
| 3 | 139La | CD66b | 24 | 161Dy | CD183[CXCR3] |
| 4 | 141Pr | CD56 | 25 | 162Dy | FoxP3 |
| 5 | 142Nd | TCRgd | 26 | 163Dy | CD33 |
| 6 | 143Nd | CD196[CCR6] | 27 | 164Dy | CD141 |
| 7 | 144Nd | CD14 | 28 | 165Ho | CD303 |
| 8 | 145Nd | CD95[Fas] | 29 | 166Er | Perforin |
| 9 | 146Nd | CD123 | 30 | 167Er | CD45RA |
| 10 | 147Sm | CD197[CCR7] | 31 | 168Er | CD11c |
| 11 | 148Nd | NKG2A | 32 | 169Tm | CD45RO |
| 12 | 149Sm | CD62L | 33 | 170Er | CD127 |
| 13 | 150Nd | CD1c | 34 | 171Yb | CD39 |
| 14 | 151Eu | CD38 | 35 | 172Yb | CD19 |
| 15 | 152Sm | CD27 | 36 | 173Yb | Granzyme B |
| 16 | 153Eu | CD69 | 37 | 174Yb | CD279[PD1] |
| 17 | 154Sm | CD152[CTLA4] | 38 | 175Lu | CD7 |
| 18 | 155Gd | KLRG1 | 39 | 176Yb | HLA-DR |
| 19 | 156Gd | NKG2D | 40 | 197Au | CD4 |
| 20 | 157Gd | NKG2C | 41 | 198Pt | CD8a |
| 21 | 158Gd | CD86 | 42 | 209Bi | CD11b |

NOTO: CyTOF: mass cytometry

Table s3. The classification of all CD45^+^ immune cells and the key marker expression

|  | **cell type** | **subtype1** | **subtype2** | **key marker expression** |
| --- | --- | --- | --- | --- |
| C01 | Unknown |  |  | CD11b^+^ |
| C02 | cDC |  |  | CD11c^+^ HLA^-^DR^+^ CD11b^-^ CD14^-^ CD1c^+^ |
| C03 | Monocytes |  |  | CD14^+^ HLA^-^ DR^+^ CD11b^+^ |
| C04 | Monocytes |  |  | CD14^+^ HLA^-^ DR^+^ CD11b^+^ CCR6^+^ |
| C05 | pDC |  |  | CD123^+^ HLA^-^ DR^+^ |
| C06 | B cells | CD11c^+^ B |  | CD19^+^ HLA^-^ DR^+^ CD11b^-^ CD11c^+^ CD303^+^ |
| C07 | B cells |  |  | CD19^+^ HLA^-^ DR^+^ CD11b^-^ |
| C08 | B cells | Plasmablasts |  | CD19^+^ HLA^-^ DR^+^ CD11b^-^ CD27^+^ CD38^+^ |
| C09 | T cells | DNT |  | CD3^+^ CD4^-^ CD8^-^ CD27^+^ CD38^+^ |
| C10 | Unknown |  |  | CD69^+^ CD38^+^ CD141^+^ |
| C11 | Basophils |  |  | CD123^+^ HLA^-^DR^-^ CD25^+^ CD11c^+^ |
| C12 | Basophils |  |  | CD123^+^ HLA^-^ DR^-^ |
| C13 | NK cells |  |  | CD56highCD16^-^ Perforin^-^ Granzyme B^-^ |
| C14 | NK cells |  |  | CD56^+^ Perforin^+^ Granzyme B^+^ |
| C15 | NK cells |  |  | CD56^+^ KLRG1^+^ NKG2C^+^ Perforin^+^ Granzyme B^+^ CD11c^-^ |
| C16 | NK cells |  |  | CD56^+^ NKG2C^+^ Perforin^+^ Granzyme B^+^ |
| C17 | T cells | CD4^+^ T | Central memory | CD3^+^ CD4^+^ CD45RA^-^ CCR7^+^ |
| C18 | T cells | CD4^+^ T | Naïve | CD3^+^ CD4^+^ CD45RA^+^ CCR7^+^ CD62L^+^ CD38^+^ |
| C19 | T cells | CD4^+^ T | Naïve | CD3^+^ CD4^+^ CD45RA^+^ CCR7^+^ |
| C20 | T cells | CD4^+^ T | Effector memory | CD3^+^ CD4^+^ PD1^+^ CD45RA^-^ CCR7^-^ |
| C21 | T cells | CD4^+^ T | Treg | CD3^+^ CD4^+^ CD25^+^ CD127^-^ |
| C22 | T cells | CD4^+^ T | Treg | CD3^+^ CD4^+^ CD25^+^ CD127^-^ CD39^+^ |
| C23 | T cells | CD4^+^ T | Central memory | CD3^+^ CD4^+^ CD45RA^-^ CCR7^+^ CD127^+^ CD27^+^ |
| C24 | T cells | CD4^+^ T | Effector memory | CD3^+^ CD4^+^ CD45RA^-^ CCR7^-^ CD7^-^ |
| C25 | T cells | CD4^+^ T | Effector memory | CD3^+^ CD4^+^ CD45RA^-^ CCR7^-^CD27^+^ KLRG1^+^ |
| C26 | T cells | CD4^+^ T | Central memory | CD3^+^ CD4^+^ CD45RA^-^ CCR7^+^ CD127^+^ |
| C27 | T cells | CD4^+^ T | Effector memory | CD3^+^ CD4^+^ CD45RA^-^ CCR7^-^ |
| C28 | T cells | gdT |  | CD3^+^ TCRgd^+^ CD56^+^ Granzyme B^+^ Perforin^+^ |
| C29 | T cells | gdT |  | CD3^+^ TCRgd^+^ Granzyem B low Perforin low |
| C30 | T cells | CD8^+^ T | Effector/NKT | CD3^+^ CD8^+^ CD56^+^ CD69^+^ CD11c^+^ CD45RA^+^ CCR7^-^ |
| C31 | T cells | CD8^+^ T | Effector memory | CD3^+^ CD8^+^ CD45RA^-^ CCR7^-^ Granzyme_B^+^ |
| C32 | T cells | CD8^+^ T | Effector | CD3^+^ CD8^+^ CD45RA^+^ CCR7^-^ |
| C33 | T cells | CD8^+^ T | Naïve | CD3^+^ CD8^+^ CD45RA^+^ CCR7^+^ |
| C34 | T cells | CD8^+^ T | Naïve | CD3^+^ CD8^+^ CD45RA^+^ CCR7^+^ |
| C35 | T cells | CD8^+^ T | Effector memory | CD3^+^ CD8^+^ PD1^+^ CD45RA^-^ CCR7^-^ |
| C36 | T cells | CD8^+^ T | Effector memory | CD3^+^ CD8^+^ CD45RA^-^ CCR7^-^ |
| C37 | T cells | CD8^+^ T | Effector memory | CD3^+^ CD8^+^ CD45RA^-^ CCR7^-^KLRG1^+^ |

NOTO: cDC = conventional dendritic cell, pDC = plasmacytoid dendritic cell, NK = natural killer

**Supplementary Figure:**


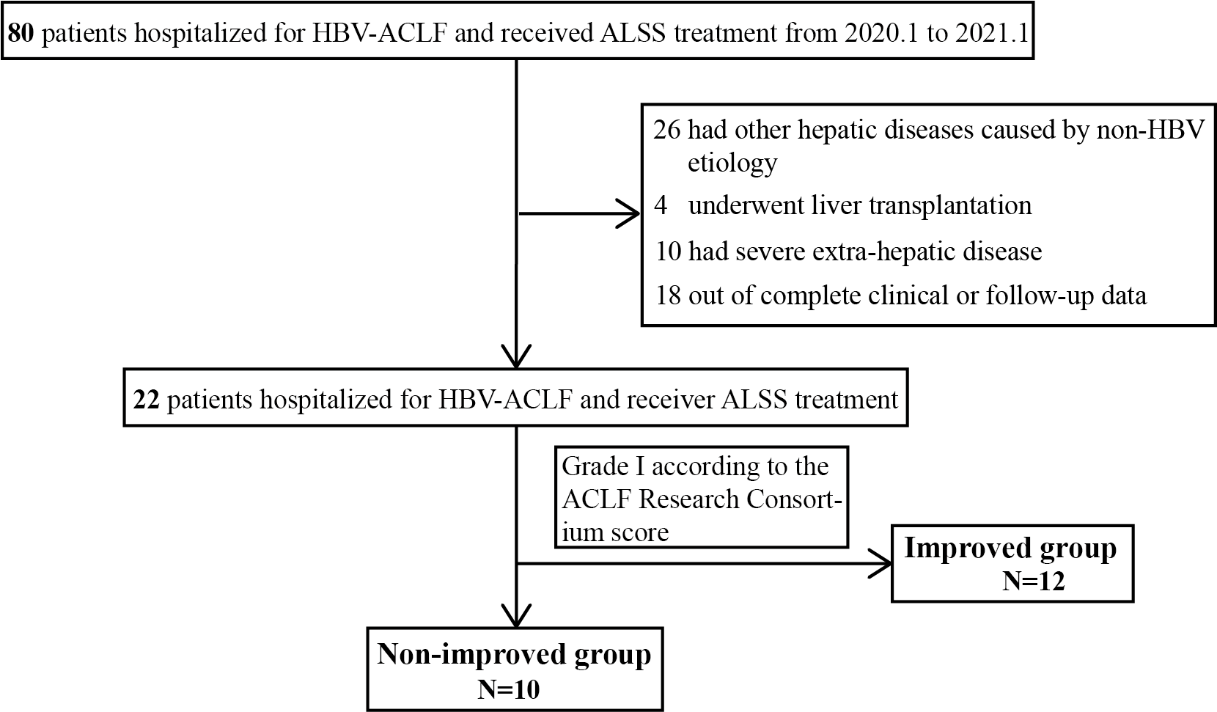


**Fig s1: Flow chart of inclusion of HBV-ACLF patients treated with ALSS.** HBV-ACLF: hepatitis B virus-related acute-on-chronic liver failure; ALSS: artificial liver support system.


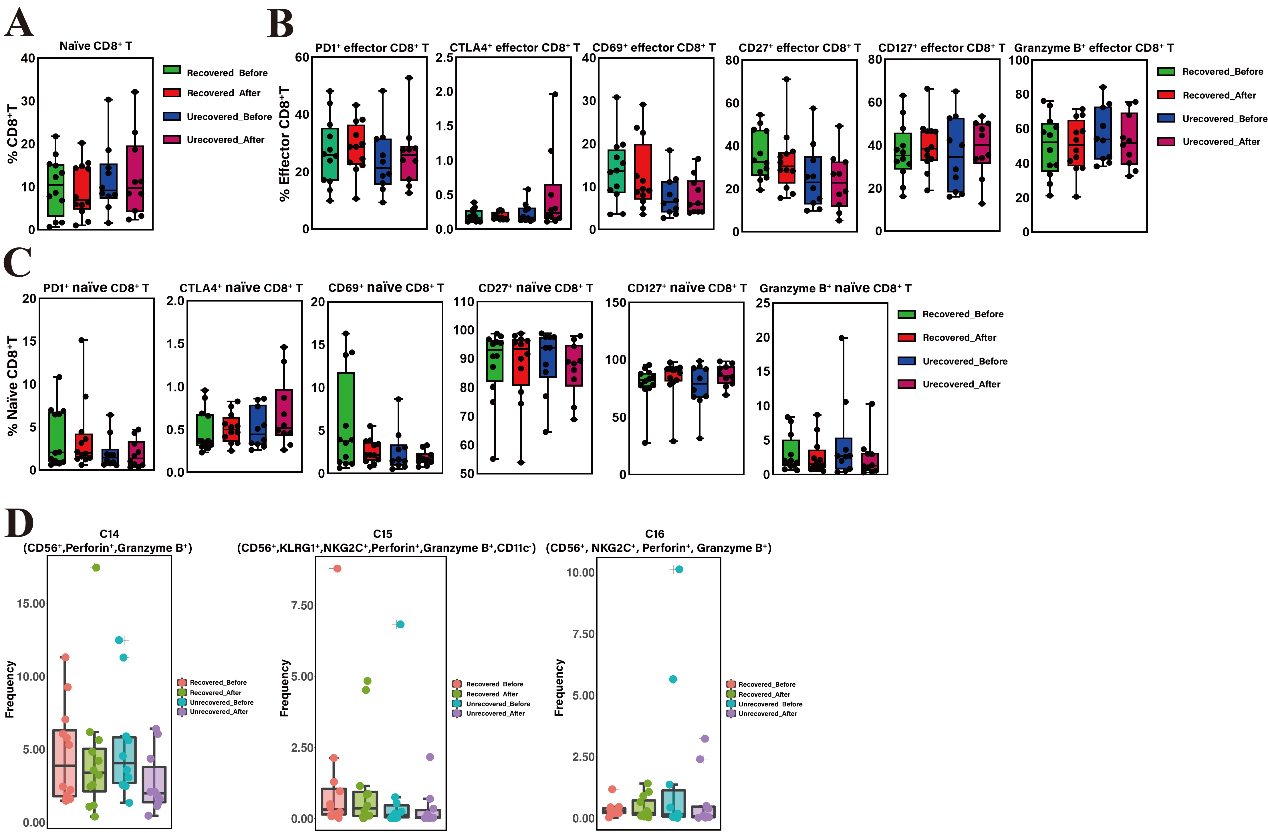


**Fig s2: A.** The proportion of naïve CD8^+^ T between improved and non-improved patients before and after ALSS therapy. **B.** The expression of PD1, CTLA4, CD69, CD27, CD127, and Granzyme B on effector CD8^+^ T cells in the two groups before and after treatment. **C.** The expression of PD1, CTLA4, CD69, CD27, CD127, and Granzyme B on naïve CD8^+^ T cells in the two groups before and after treatment. **D.** The frequency of C14, C15, and C16 (NK cells) clusters between improved and non-improved patients before and after ALSS therapy. NK: natural kill; ALSS: artificial liver support system.

**
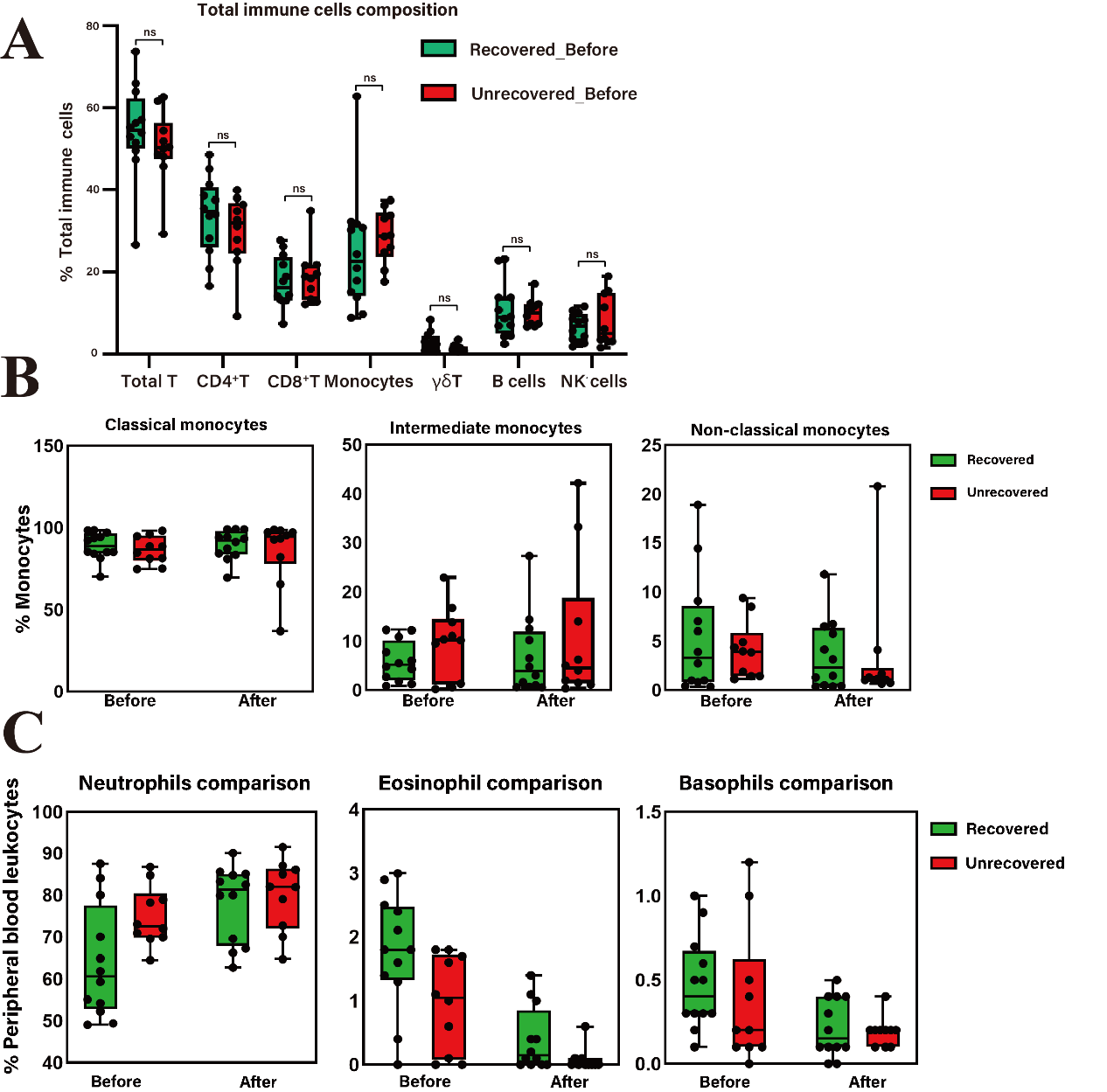
**

**Fig s3: A.** Boxplots displaying the difference between immune cell subsets of two groups in total immune cell level before ALSS therapy. **B.** The proportion of classical, intermediate, and non-classical monocytes between improved and non-improved patients before and after ALSS therapy. **C.** The changes of granulocytes in peripheral blood of two groups before and after treatment. ALSS: artificial liver support system.
