# Supplementary figures and images for: Characteristics of circulating immune cells in HBV-related acute-on-chronic liver failure following artificial liver treatment
Source: BMC Immunol. 2023 Nov 25;24:47. doi: 10.1186/s12865-023-00579-8 (PMC10676598; doi:10.1186/s12865-023-00579-8)

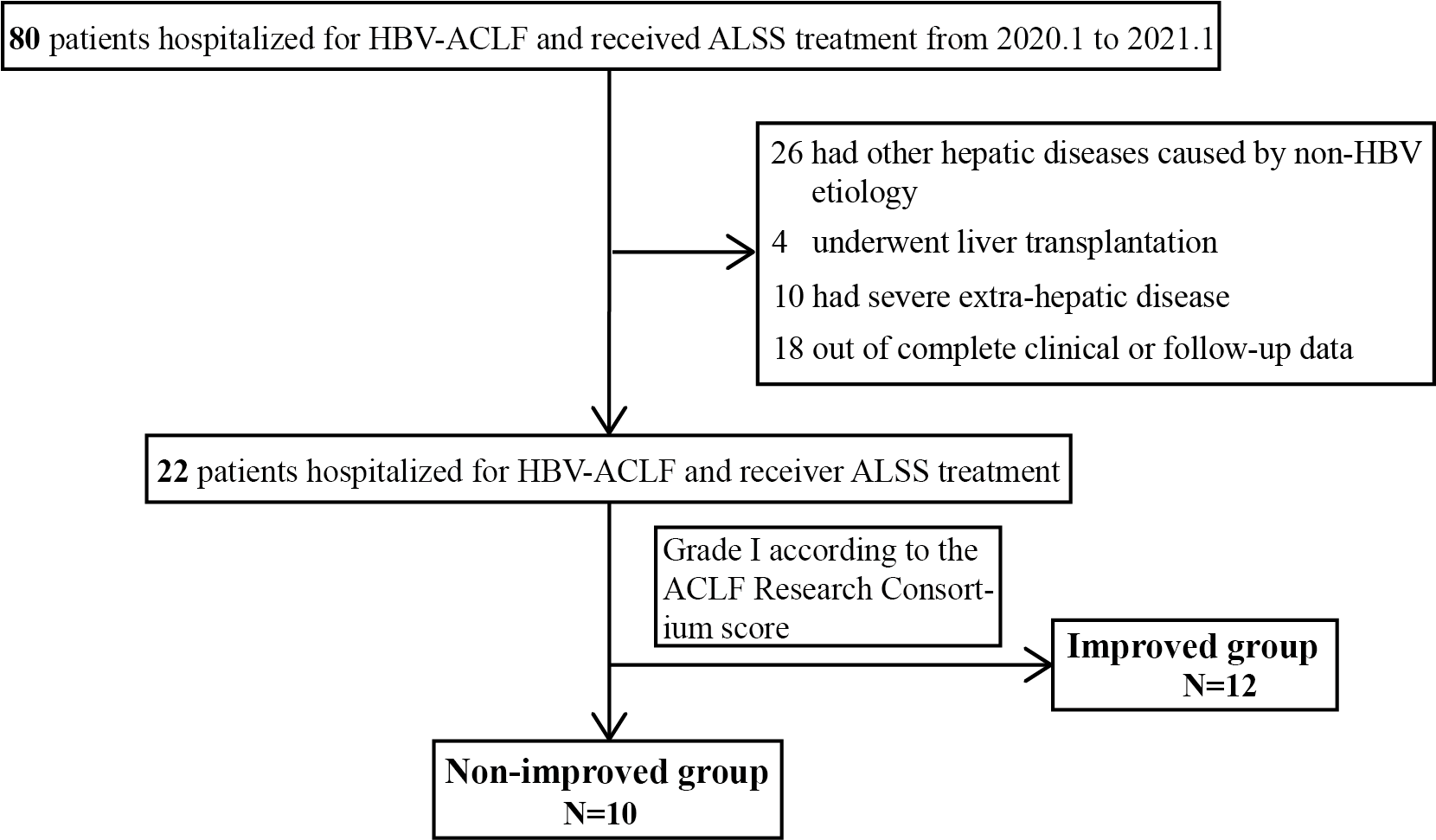

Supplement: Supplementary file 1 — Additional file 1: Table S1. Cryopreservation and resuscitation of PBMCs. Table S2. CyTOF panel. Table S3. The classification of all CD45+ immune cells and the key marker expression. Fig. S1. Flow chart of inclusion of HBV-ACLF patients treated with ALSS. HBV-ACLF: hepatitis B virus-related acute-on-chronic liver failure; ALSS: artificial liver support system. Fig. S2. A. The proportion of naïve CD8+ T between improved and non-improved patients before and after ALSS therapy. B. The expression of PD1, CTLA4, CD69, CD27, CD127, and Granzyme B on effector CD8+ T cells in the two groups before and after treatment. C. The expression of PD1, CTLA4, CD69, CD27, CD127, and Granzyme B on naïve CD8+ T cells in the two groups before and after treatment. D. The frequency of C14, C15, and C16 (NK cells) clusters between improved and non-improved patients before and after ALSS therapy. NK: natural kill; ALSS: artificial liver support system. Fig. S3. A. Boxplots displaying the difference between immune cell subsets of two groups in total immune cell level before ALSS therapy. B. The proportion of classical, intermediate, and non-classical monocytes between improved and non-improved patients before and after ALSS therapy. C. The changes of granulocytes in peripheral blood of two groups before and after treatment. ALSS: artificial liver support system. [file 12865_2023_579_MOESM1_ESM.zip › Supplementary Fig 1.tif]

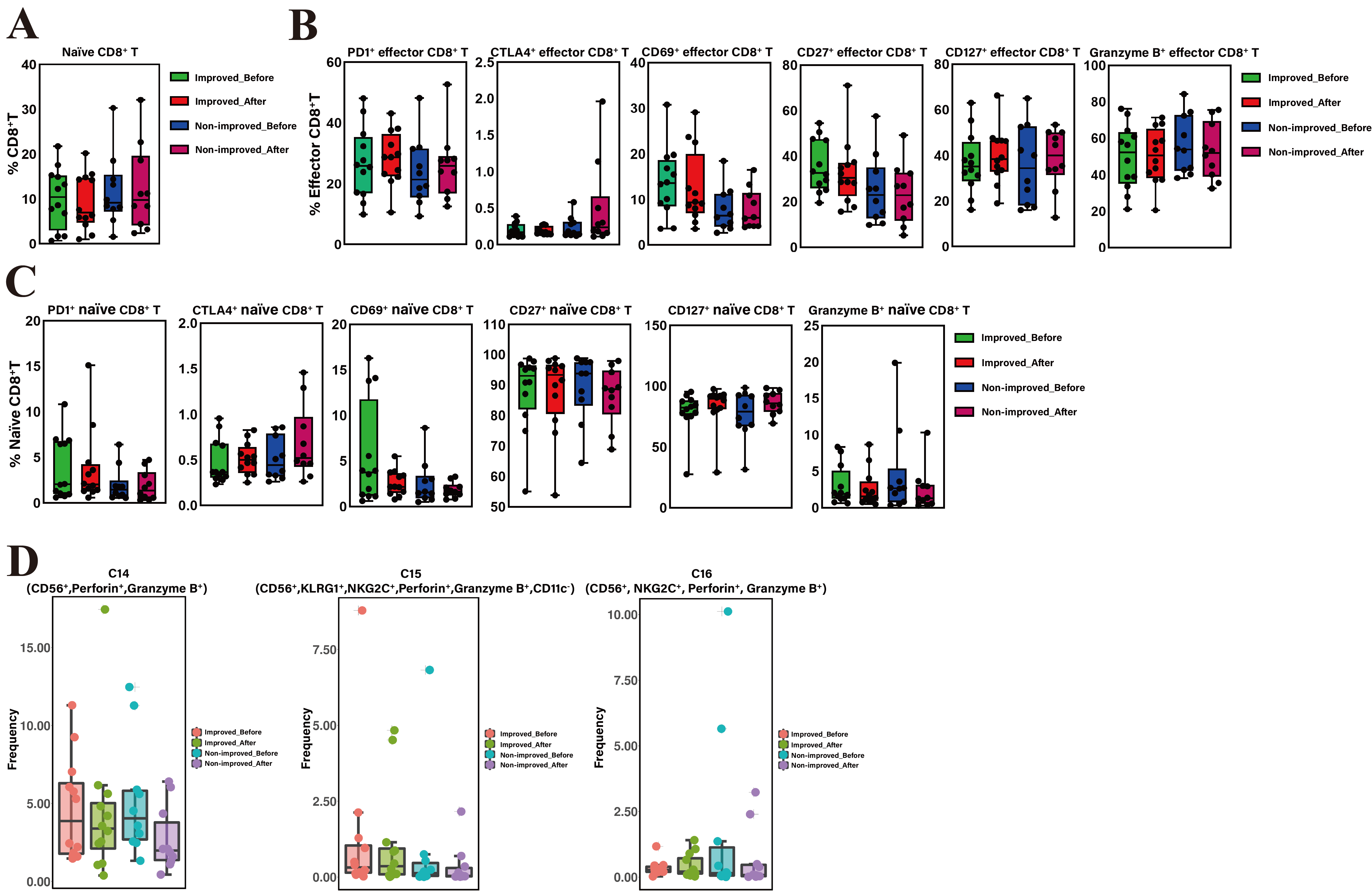

Supplement: Supplementary file 1 — Additional file 1: Table S1. Cryopreservation and resuscitation of PBMCs. Table S2. CyTOF panel. Table S3. The classification of all CD45+ immune cells and the key marker expression. Fig. S1. Flow chart of inclusion of HBV-ACLF patients treated with ALSS. HBV-ACLF: hepatitis B virus-related acute-on-chronic liver failure; ALSS: artificial liver support system. Fig. S2. A. The proportion of naïve CD8+ T between improved and non-improved patients before and after ALSS therapy. B. The expression of PD1, CTLA4, CD69, CD27, CD127, and Granzyme B on effector CD8+ T cells in the two groups before and after treatment. C. The expression of PD1, CTLA4, CD69, CD27, CD127, and Granzyme B on naïve CD8+ T cells in the two groups before and after treatment. D. The frequency of C14, C15, and C16 (NK cells) clusters between improved and non-improved patients before and after ALSS therapy. NK: natural kill; ALSS: artificial liver support system. Fig. S3. A. Boxplots displaying the difference between immune cell subsets of two groups in total immune cell level before ALSS therapy. B. The proportion of classical, intermediate, and non-classical monocytes between improved and non-improved patients before and after ALSS therapy. C. The changes of granulocytes in peripheral blood of two groups before and after treatment. ALSS: artificial liver support system. [file 12865_2023_579_MOESM1_ESM.zip › Supplementary Fig 2.tif]

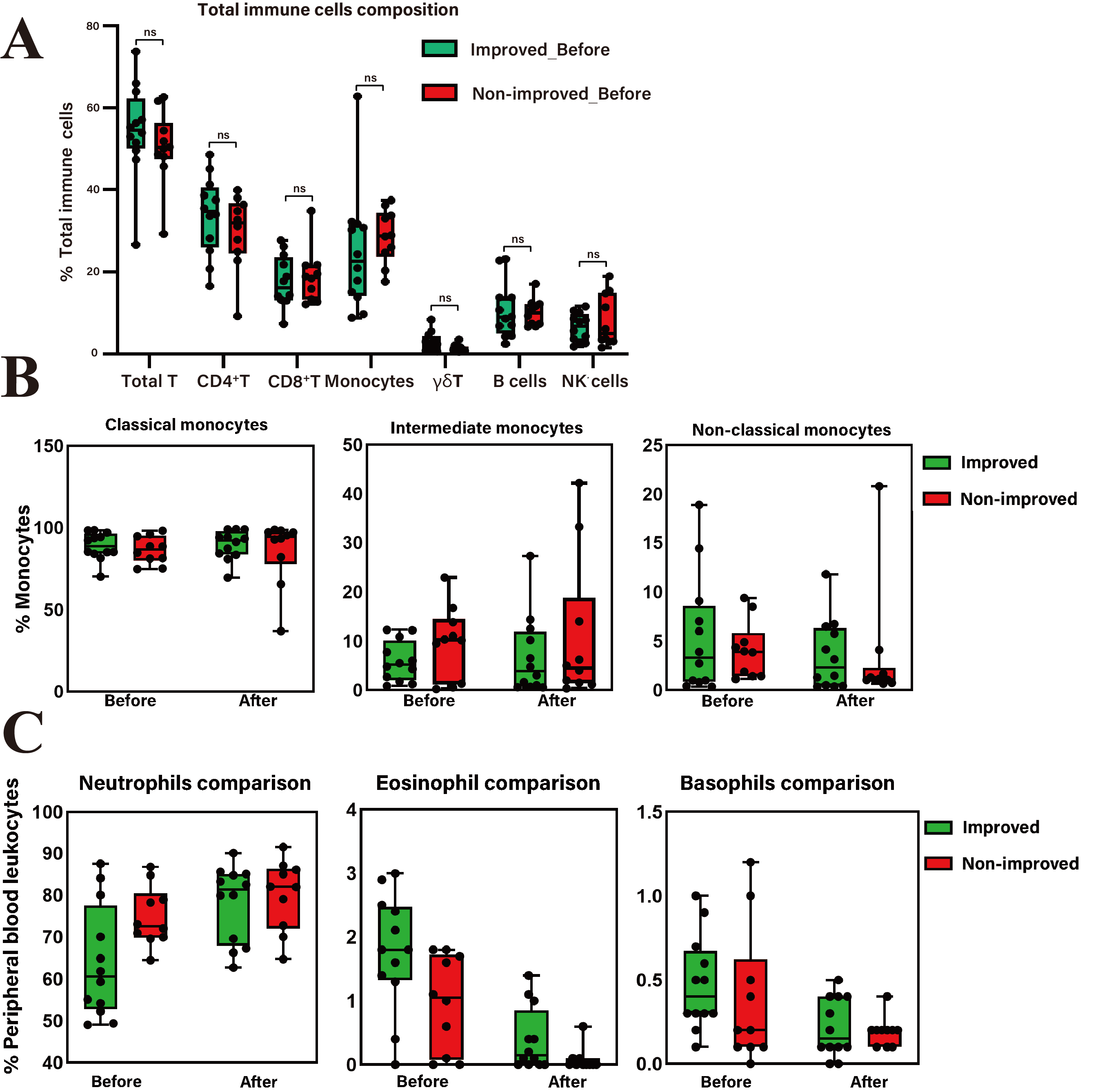

Supplement: Supplementary file 1 — Additional file 1: Table S1. Cryopreservation and resuscitation of PBMCs. Table S2. CyTOF panel. Table S3. The classification of all CD45+ immune cells and the key marker expression. Fig. S1. Flow chart of inclusion of HBV-ACLF patients treated with ALSS. HBV-ACLF: hepatitis B virus-related acute-on-chronic liver failure; ALSS: artificial liver support system. Fig. S2. A. The proportion of naïve CD8+ T between improved and non-improved patients before and after ALSS therapy. B. The expression of PD1, CTLA4, CD69, CD27, CD127, and Granzyme B on effector CD8+ T cells in the two groups before and after treatment. C. The expression of PD1, CTLA4, CD69, CD27, CD127, and Granzyme B on naïve CD8+ T cells in the two groups before and after treatment. D. The frequency of C14, C15, and C16 (NK cells) clusters between improved and non-improved patients before and after ALSS therapy. NK: natural kill; ALSS: artificial liver support system. Fig. S3. A. Boxplots displaying the difference between immune cell subsets of two groups in total immune cell level before ALSS therapy. B. The proportion of classical, intermediate, and non-classical monocytes between improved and non-improved patients before and after ALSS therapy. C. The changes of granulocytes in peripheral blood of two groups before and after treatment. ALSS: artificial liver support system. [file 12865_2023_579_MOESM1_ESM.zip › Supplementary Fig 3.tif]
